# Central Venous Catheter Misplacement Into Pleural Cavity Causing Hypertensive Pleural Effusion: A Case Report

**DOI:** 10.7759/cureus.61579

**Published:** 2024-06-03

**Authors:** Sancha Costa Santos, Helena Silva, Joana Varandas, Grimanesa Sousa, Rui Silva

**Affiliations:** 1 Anaesthesiology, Hospital do Divino Espírito Santo de Ponta Delgada, EPE, Ponta Delgada, PRT; 2 Intensive Care Unit, Hospital do Divino Espírito Santo de Ponta Delgada, EPE, Ponta Delgada, PRT; 3 Anaesthesiology, Hospital do Divino Espírito Santo de Ponta Delgada, EPE, Ponta Delgada , PRT

**Keywords:** pleural cavity, cardiopulmonary resuscitation, cardiorespiratory arrest, fluids, shock, central venous catheter

## Abstract

Central venous catheter (CVC) insertion is a routine procedure in the management of critically ill patients. We report a clinical case of inadvertent placement of an internal jugular vein CVC into the right pleural cavity, despite employing clinical and imaging-based techniques to ensure proper catheter positioning. Infusion of fluids and vasopressors through this misplaced catheter led to hypertensive pleural effusion and subsequent cardiorespiratory arrest. Return of spontaneous circulation was achieved after two cycles of cardiopulmonary resuscitation. While multiple imaging modalities are recommended for confirming appropriate CVC placement, each method has inherent limitations. This case highlights the imperative need for a high index of suspicion to avert such complications and pretends to review some of each method’s limitations.

## Introduction

A central venous catheter (CVC) is a device essential in medical practice, commonly positioned in central veins, particularly the superior vena cava (SVC) or cavo-atrial junction (CAJ) [1]. It serves multiple purposes, including central venous pressure monitoring, administration of intravenous fluids, vasopressors, and nutrition [2]. Several anatomical sites can provide central venous access, such as the internal jugular vein (IJV), subclavian vein, or femoral vein. IJV insertion is frequently preferred due to the ease of use of ultrasound guidance and the decreased rate of catheter insertion failure and malposition. Additionally, the carotid artery is more compressible than the subclavian artery in case of arterial puncture [3].

Previous studies have reported varying frequencies of mechanical complications in CVC insertion, ranging from 1.1% to 18.8% [4]. Complications arising from the insertion and maintenance of a CVC include unintended arterial puncture, catheter malposition, pneumothorax, chylothorax, accidental dislodgment, occlusive thrombosis, and infection [4,5].

Guidelines recommend various techniques for safe CVC placement, including ultrasound guidance, monitoring central pressure waveform, blood gas analysis, electrocardiogram (EKG) guidance, and chest X-ray (CXR) to assess complications and line positioning [1-3].

While improvements in placement techniques have lowered complication rates, documenting such occurrences in literature remains crucial to raising awareness among clinicians. We report a case of a hypertensive pleural effusion resulting from CVC misplacement in the right pleural cavity, leading to cardiorespiratory arrest and successful return of spontaneous circulation (ROSC) following two cycles of cardiopulmonary resuscitation cycles (CPR). Informed consent was obtained from the family for this case. This article was previously presented as an oral communication at “O Norte da Anestesia 2022 - Porto Anaesthesiology International Congress” on November 19, 2022.

## Case presentation

A 47-year-old man was admitted to the emergency department (ED) with a 10-day history of diarrhea, abdominal pain, and fever. Previous medical history included arterial hypertension, chronic obstructive pulmonary disease (COPD), tobacco and alcohol abuse, and alcoholic liver disease. On admission, the patient exhibited a Glasgow coma scale of 12, pulse oximetry of 95% (fraction of inspired oxygen of 21%), heart rate of 112 bpm, blood pressure of 86/46 mmHg, temperature of 38.8°C, and a lower abdominal pain without rebound tenderness. Laboratory investigations revealed liver and renal dysfunction, abnormal inflammatory markers, and lactates of 2.10 mg/dL.

**Figure 1:**
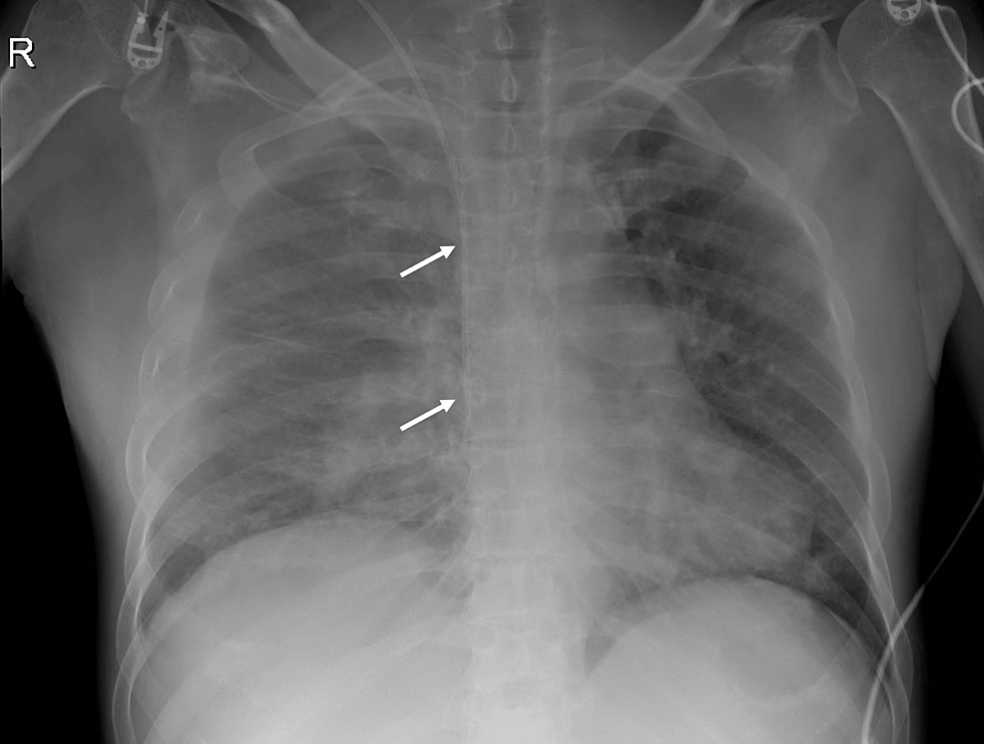
Anteroposterior CXR after CVC placement (white arrows show CVC location) CXR, chest X-ray; CVC, central venous catheter

Abdominal ultrasound confirmed severe liver steatosis with medium-volume ascites, and abdominal computed tomography angiography (CTA) scan demonstrated signs of colorectal colitis. Septic shock secondary to colorectal colitis was diagnosed, prompting initiation of empiric antibiotic with piperacillin-tazobactam 4.5 g. In the ED, a three-lumen 20 cm CVC was inserted into the right IJV to start intensive fluid therapy and vasopressor administration. CVC was introduced using the Seldinger technique under ultrasound guidance, yet the initial two attempts were unsuccessful due to encountered resistance while advancing the J-tip guidewire. Despite experiencing some resistance during the third attempt at guidewire advancement, distinct characteristic changes in the P-wave were observed. Besides, aspiration and backflow of blood through the three ports and flush of all three lumens with normal saline were possible. Posteroanterior CXR showed the catheter orientated vertically over the anatomical location of the SVC, with its tip positioned at the level of the CAJ, approximately two vertebral bodies below the carina’s level, as shown in Figure 1.

**Figure 2:**
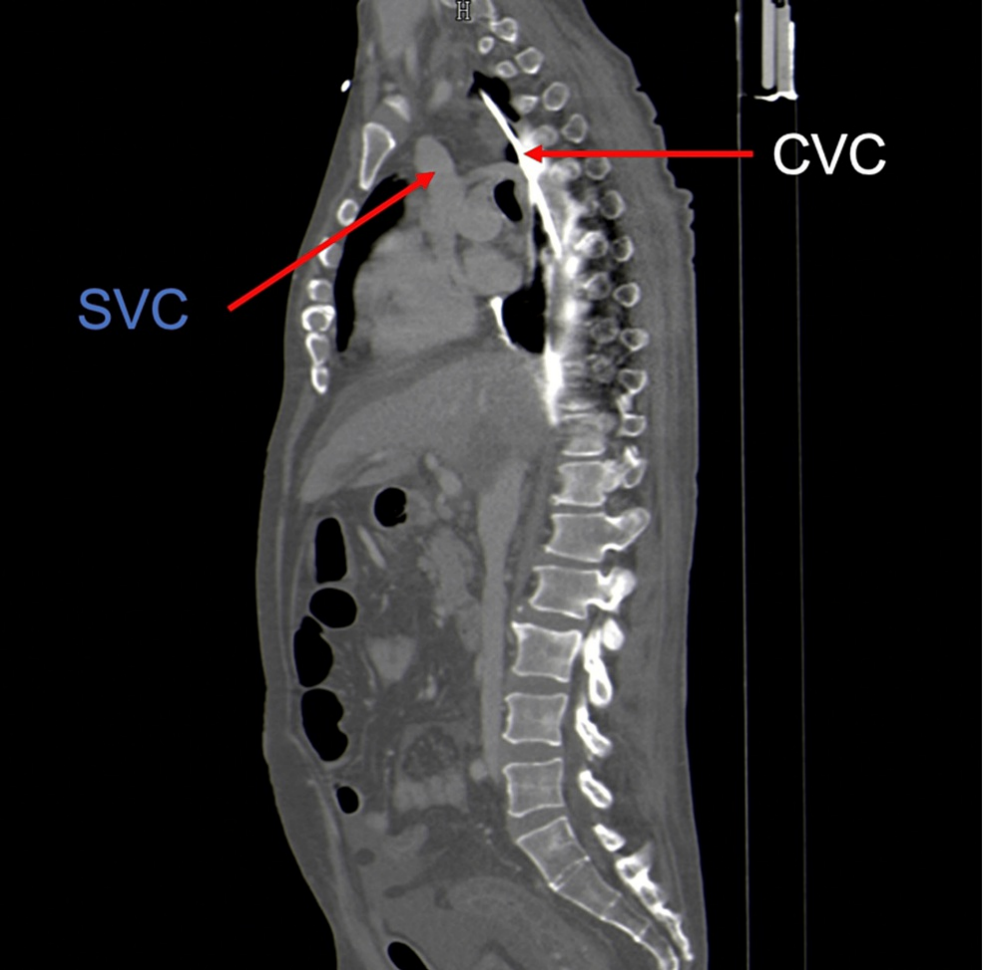
CTA scan showing extravascular CVC path along the posterior border of the SVC (sagittal view) CTA, computed tomography angiography; CVC, central venous catheter; SVC, superior vena cava

**Figure 3:**
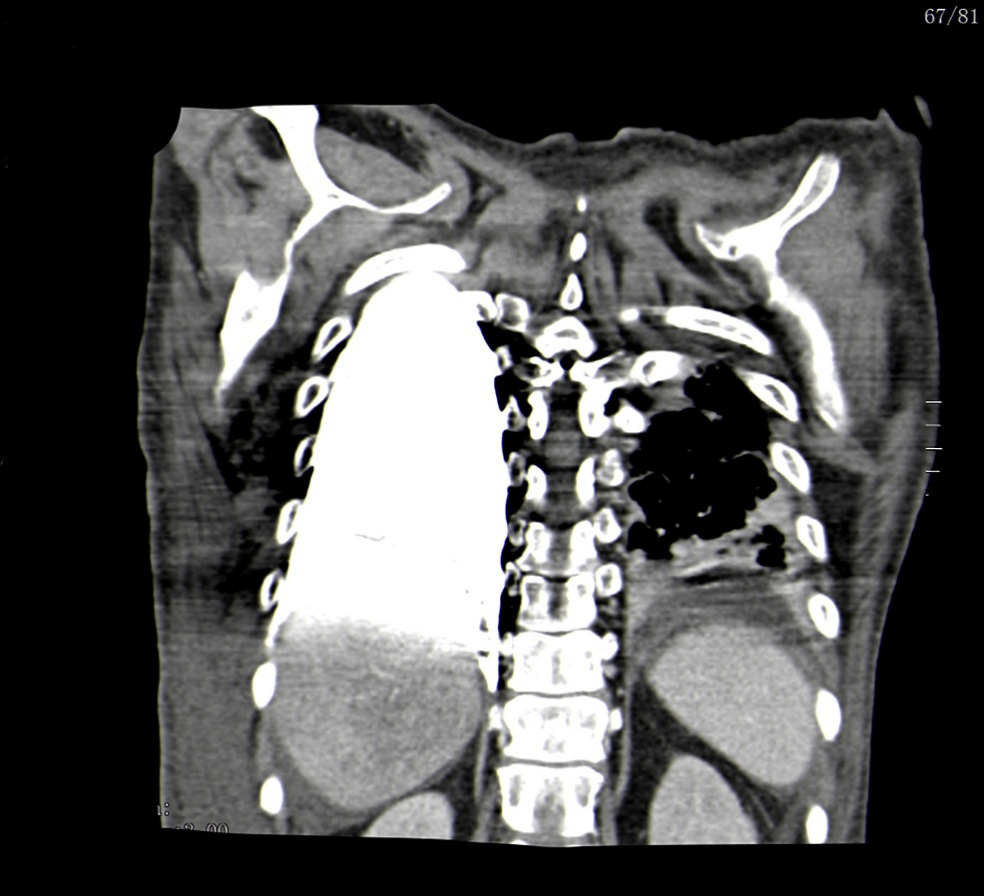
CTA scan showing contrast spreading through pleural space (coronal view) CTA, computed tomography angiography

The catheter was thought to be correctly positioned, prompting the initiation of fluids, vasopressor, and antibiotic administration through it. He remained hypotensive and oliguric despite vigorous fluid therapy and vasopressor support. A few hours later, he presented with respiratory distress, marked by dyspnea, wheezing, diminished breath sounds in bilateral lung fields on pulmonary auscultation, and refractoriness to bronchodilator treatment. There was progressive clinical deterioration with pallor, diaphoresis, and bradycardia with progressive altered mental status, necessitating prompt endotracheal intubation. Following intubation, the patient experienced a non-shockable rhythm cardiorespiratory arrest, with ROSC after two CPR cycles and the administration of adrenaline 1 mg. An urgent bedside thoracic ultrasound revealed a massive right pleural effusion. Subsequent drainage of approximately 4 L of serosanguinous pleural fluid led to immediate clinical improvement. Pleural fluid analysis showed abundant red blood cells with a glucose level of 530 mg/dL. Suspecting pulmonary thromboembolism, CTA was performed, revealing an extravascular CVC path along the posterior border of the SVC, with the catheter tip ending in the pleural cavity and contrast dispersion through this space, as shown in Figures 2, 3.

**Figure 4:**
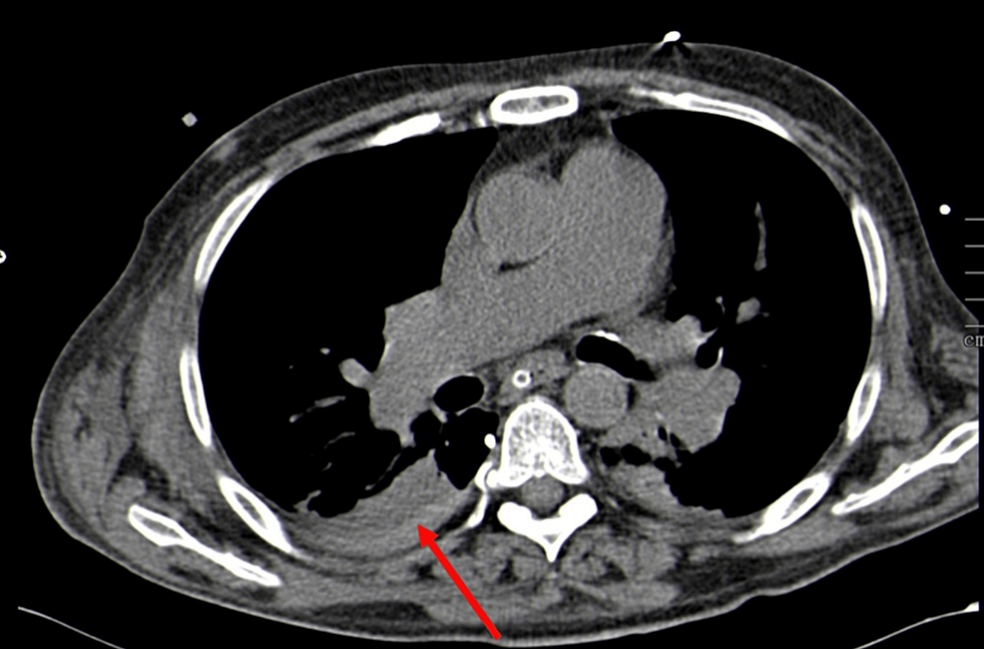
CTA scan showing hemothorax (red arrow) in the right pleural cavity (axial view) CTA, computed tomography angiography

Additionally, a mild medial pneumothorax and a hemothorax were observed, which is seen in Figure 4.

The CVC was safely removed and replaced in the left IJV. The patient was subsequently transferred to the intensive care unit (ICU), exhibiting clinical improvement, with removal of the thoracic drain on the third day and successful extubation on the fourth day of mechanical ventilation. A follow-up CXR demonstrated significant improvement in right lung opacification with no evidence of pleural effusion or pneumothorax. The patient was discharged to the internal medicine ward with progressive clinical improvement in the first week. However, during the second week, the patient developed pneumonia caused by *Klebsiella pneumoniae*, which led to a fatal outcome.

## Discussion

Central venous catheterization is frequently necessary to assist critically ill patients admitted to the ED and ICU. CVC placement is associated with potential immediate or delayed complications, including mechanical, infectious, or thrombotic events [4,5]. We report a mechanical complication during CVC insertion, namely an extravascular cannulation, resulting in a hypertensive pleural effusion and a mild pneumothorax and hemothorax. 

The hypertensive pleural effusion was caused by all the fluids and vasopressors being infused through the CVC. Tension physiology occurred with the progressive accumulation of fluids in the pleural space and increasing positive pressure within the chest, with compression of the ipsilateral and contralateral lung, trachea, heart, and vena cava, causing inadequate preload and obstructive shock. 

The proximity of the SVC to the mediastinal pleura in the upper thorax increases the risk of uncontrolled bleeding into the low-pressure pleural space when there’s a vein wall perforation during CVC insertion [2]. We consider that perforation of the SVC during the initial two attempts might explain the subsequent hemothorax observed in CTA. This hypothesis is supported by the patient’s presentation without respiratory symptoms upon ED admission and the absence of pleural effusion or hemothorax on the initial CXR. Moreover, initial attempts might have created a false trajectory, which may explain the absence of resistance during the third CVC placement attempt. The aspirated blood was retrospectively considered to be the hemothorax in the right pleural cavity. 

While pleural effusion development following misplaced CVC placement has been previously described, most cases involve delayed catheter migrations or early malposition diagnoses based on unsuccessful blood aspiration or misaligned catheter visualized in follow-up CXRs [6,7]. Our case reports a total extravascular CVC misplacement not immediately detected as the puncture was ultrasound-guided, there was blood aspiration in the three ports, and CXR showed the catheter to be apparently correctly positioned. 

Ultrasound guidance is valuable for initial vessel puncture but is of limited value in confirming the tip’s position within the SVC. Additionally, EKG guidance helps verify the central placement of catheters within the chest at the CAJ. Yet, characteristic P-wave changes may occur whenever a guidewire or conducting catheter is close to the right or left atrium, irrespective of whether it is in a vein, artery, mediastinum, or other structure [2]. While radiographs aid in confirming catheter position and detecting complications post-CVC insertion, limitations persist due to their two-dimensional projection and the involved anatomical arrangement of major vessels and pleura in the neck and chest [2]. In our case, despite the apparent alignment of the CVC with the SVC on the CXR, it followed a posterior path outside the vessel. 

Lockwood and Desai report the steps for a correct insertion technique of a CVC [3]. In line with this evidence, our case failed to confirm the guidewire’s correct venous position in the ultrasound long axis views, and central venous pressure waveform monitoring was not observed, which could have immediately shown a waveform inconsistent with vascular placement. Blood gas analysis after CVC insertion would not have distinguished a correct insertion from a malposition in the context of hemothorax. 

Despite initial clinical improvement, the patient unfortunately died in the context of respiratory failure due to nosocomial pneumonia caused by *K. pneumoniae*. Several factors potentially contributed to this outcome. The patient presented several risk factors for *K. pneumoniae* infection, including COPD, alcoholism, and hepatobiliary disease [8]. Additionally, the use of invasive medical devices, such as intravenous catheters, represents a significant risk factor for such infections [8], and intubation itself is the most significant risk factor for the development of hospital-acquired pneumonia, particularly in a patient with a personal history of COPD [9]. Whereas initial tube thoracostomy may effectively drain liquid blood from the pleural space, clotted or loculated collections of blood may not be evacuated by single or even multiple chest tubes. Retained blood in the pleural space is a risk factor for the development of further complications, including empyema [10]. The extent to which this incident influenced the outcome remains uncertain.

## Conclusions

In conclusion, though many techniques and imaging modalities are recommended to confirm appropriate CVC placement, each has its limitations with potentially disastrous consequences. Rapid respiratory deterioration and accumulating pleural effusion following difficult cannulation should raise suspicion of a misplaced CVC in the pleural cavity. Healthcare practitioners should be diligent during CVC insertion and remain vigilant regarding potential post-insertion complications, even after seemingly correctly positioned catheters.
